# Ezetimibe and Improving Cardiovascular Outcomes: Current Evidence and Perspectives

**DOI:** 10.1155/2020/9815016

**Published:** 2020-06-28

**Authors:** Akshyaya Pradhan, Monika Bhandari, Rishi Sethi

**Affiliations:** Department of Cardiology, King George's Medical University, Lucknow, Uttar Pradesh 226003, India

## Abstract

Low-density lipoprotein lowering with statins has convincingly and consistently proven to reduce cardiovascular events in both primary and secondary prevention. However, despite high-dose statin therapy, residual cardiovascular risk remains and many patients also do not tolerate statins. Ezetimibe was initially projected as a frontline alternative to statin. It is an intestinal cholesterol absorption inhibitor with modest LDL lowering effects. But, major studies failed to demonstrate any beneficial effect of CV outcomes, and the drug was relegated to oblivion. IMPROVE-IT, a contemporary, large, and well-designed trial, unequivocally demonstrated reduction in CV outcomes with ezetimibe when added to statin therapy. The benefits are seen in both sexes, elderly, CKD, diabetes mellitus, and in patients with prior CABG. It also reduces biomarkers and induces plaque regression like statins. The drug has now established itself as an add-on therapy to statin when monotherapy fails to achieve LDL goals and when it is not tolerated. The combination therapy has excellent safety and efficacy record. It has now been endorsed by major guidelines too in management of dyslipidemia. Yes, ezetimibe can indeed improve cardiovascular outcomes!

## 1. Introduction

It is well known fact that deposition of LDL-C and cholesterol-rich Apo-B containing lipoproteins in the arterial walls is responsible for atherosclerosis. Atherosclerotic cardiovascular diseases (ASCVD include patients with acute coronary syndromes, prior myocardial infarction (MI), stable or unstable angina, history of past arterial revascularization, stroke, transient ischemic attack (TIA), or peripheral artery disease (PAD) including aortic aneurysm, all of atherosclerotic origin) are the major cause of death worldwide, so prevention of ASCVD by control of risk factors including hypercholesterolemia, diabetes, and hypertension is important to save lives. Plasma low-density lipoprotein cholesterol (LDL-C) is a measure of cholesterol mass carried by LDL particles. Mendelian studies and randomized control trials have consistently shown a log-linear relationship between absolute changes in plasma LDL-C and the risk of ASCVD. Also, it has been revealed in Mendelian studies that long-term exposure to low LDL-C lowers the risk for CV events.

### 1.1. LDL Lowering Reduces CV Events

Positive correlation of LDL-C and Cardiovascular (CV) risk is demonstrated by various epidemiological studies, and this relationship even extends to low LDL-C levels [1, 2]. We also know from genetic studies that an individual with lifetime exposure to high LDL-C as in heterozygous familial hypercholesterolemia is at high risk for premature atherosclerosis and cardiovascular disease [3]. On the contrary, individuals who have genetic mutations for low LDL-C have lower chances of atherosclerotic cardiovascular diseases (ASCVD) [4]. Statins are 3-hydroxy-3-methylglutaryl-coenzyme A (HMG-CoA) reductase inhibitors. A meta-analysis of 27 randomized trials involving 174,000 participants revealed that for every ∼40 mg/dL LDL-C reduction with statin therapy, the relative risk of major adverse cardiovascular events is reduced by ∼20–25%, and all-cause mortality is reduced by 10%. Intensification of statin regimens yield a 15% further reduction in major adverse cardiovascular events [5].

### 1.2. Residual Risk with Statin Therapy

**Figure 1:**
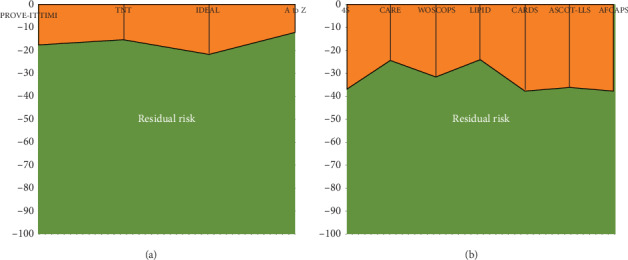
Residual risk in the large randomized statin trials for secondary (a) and primary prevention (b), respectively.

A meta-analysis of major statin trials revealed that even after intensive statin therapy, there remains a residual risk of CV events. The 5-year rate of major CV events was 22% among individuals with prior CV disease and 10% in those who did not have history of an established CV disease [6]. This residual risk may be due to lipid-related factors (high triglycerides and low high-density lipoprotein cholesterol) or nonlipid factors(inflammation). Hence, the search for nonstatin alternatives to reduce CV risk is imperative.  Figure 1 depicts the approximate residual risk in various primary and secondary prevention trials of statin therapy. In the past decade, ezetimibe has emerged as a formidable adjunct to statins in LDL lowering and CV risk reduction.

## 2. Ezetimibe: The Drug

**Figure 2:**
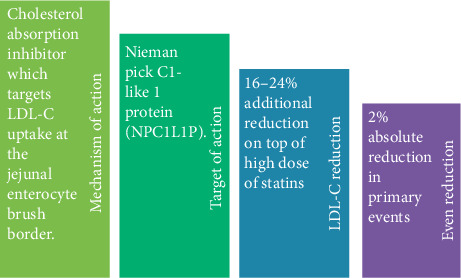
Salient features of ezetimibe.

Ezetimibe is a cholesterol absorption inhibitor, which targets LDL-C uptake at the jejunal enterocyte brush border. The primary target of action is the cholesterol transport protein Nieman Pick C1-like 1 protein (NPC1L1P). The drug is safe and well-tolerated. Statins lower the LDL-C levels by 35%–60%. But, they are associated with compensatory increase in hepatic LDL-C receptor production and enhanced uptake of serum LDL-C into the liver. Simultaneously, there is also an increased intestinal absorption of LDL-C cholesterol [7]. Thus, simultaneous use of statins and inhibitors of intestinal LDL-C absorption can yield a cumulative reduction in LDL-C in individuals with high residual LDL-C to reduce CV events [8]. The salient features of ezetimibe are summarized in Figure 2.

### 2.1. Ezetimibe: The Initial Experience Was Mixed

A positive impact of using ezetimibe on carotid atherosclerosis was observed in the Stop Atherosclerosis in Native Diabetics Study (SANDS, Table 1) [9]. A reduction in Carotid Intima-Media Thickness (CIMT) was seen in patients treated with statin and ezetimibe combination or those who received aggressive LDL-C lowering treatment. In patients who were not able to meet LDL-C targets, in multivariate analysis, it was revealed that the change in CIMT was related to the degree of LDL-C reduction and independent of specific choice of lipid-lowering therapy. Further beneficial effects of combination therapy with ezetimibe were reported in the Vytorin on and Overall Arterial Rigidity (VYCTOR) study, in which the primary end point happened to be the change in CIMT [10]. In the ezetimibe and simvastatin in the Hypercholesterolemia Enhances Atherosclerosis Regression (ENHANCE) trial [11], despite the significant difference in LDL-C lowering (−55.6% vs. −39.1%, *P* < 0.01), there was no significant difference in the average of mean carotid and femoral IMT measurements after 2 years of treatment (+0.0033 mm for placebo vs. 0.0182 mm for ezetimibe, *P* = 0.15). However, in the ARBITER 6 trial, there was insignificant reduction of CIMT with ezetimibe [12]. These negative results stood in contrast to the prior atorvastatin versus simvastatin on the Atherosclerosis Progression (ASAP) trial, in which high-dose treatment using atorvastatin 80 mg/day in subjects with heterozygous FH resulted in a greater reduction in LDL-C as compared to treatment with moderate-dose simvastatin of 40 mg/day [13]. This difference in LDL-C reduction was associated with regression of CIMT of −0.031 mm in the atorvastatin group and progression of +0.036 mm in the simvastatin group (*P* = 0.0001 for between-group comparison).

The clinical efficacy of ezetimibe was first studied in the SEAS trial. This study involved 1873 patients of mild-to-moderate aortic stenosis who were randomized to either ezetimibe 10 mg/day plus simvastatin 40 mg/day or placebo [14]. Although LDL-C was significantly reduced by 61% as compared to placebo, there was no significant reduction in composite of primary end points of need for aortic valve surgery and cardiovascular events. But, there was a significant reduction in fatal and nonfatal MI by 41% [15].

### 2.2. SHARP Trial: Beginning of Windfall for Ezetimibe

In the SHARP trial, patients of chronic kidney disease (both with or without dialysis dependence) were randomized to simvastatin 20 mg/day plus ezetimibe 10 mg/day or placebo. A significant 17% reduction in major atherosclerotic events was seen in the ezetimibe group as compared to placebo (*P* = 0.0021) after 5 years of therapy. Again, risk reduction was proportional to the magnitude of LDL-C reduction. There was no excess risk of adverse events, including myopathy and rhabdomyolysis [16].

To sum up, ezetimibe demonstrated atherosclerosis regression in SANDS and VYCTOR, while cardiovascular benefit was established in SEAS and SHARP using combination therapy with it. But, the negative outcomes reported from ENHANCE and ARBITER 6 generated significant controversy and dampened the enthusiasm for the role of ezetimibe in the treatment of hypercholesterolemia.

## 3. IMROVE-IT: The Game Changer

Despite the initial favorable outcomes with ezetimibe and statin combination, due to the dismal outcomes of ENHANCE and ARBITER 6 trials, ezetimibe was relegated from guidelines and scientific advisories. But, in 2015, the IMProved Reduction of Outcomes: Vytorin Efficacy International Trial (IMPROVE-IT) trial sorted out all the controversies and emerged as a game changer for treating hypercholesterolemic patients whose LDL-C is not controlled despite high intensity statin therapy [17].

The IMPROVE-IT trial evaluated the effect of ezetimibe in combination with simvastatin, as compared to simvastatin alone, in stable patients with recent history of an acute coronary syndrome (ACS) with LDL cholesterol within the guideline-recommended limits. Patients of 50 years of age or more with history of ACS within past 10 days with an LDL-C range of 50–100 mg/dl on statin therapy or between 50–125 mg/dl if statin naïve were included in the study. Patients were excluded if they had a creatinine clearance <30 ml/min or active liver disease or if they were planned for CABG. The primary efficacy end points of the study was a composite of death from cardiovascular death, a major coronary event (nonfatal MI, unstable angina (UA) requiring hospital admission, or coronary revascularization occurring at least 30 days after randomization), and nonfatal stroke. The key secondary efficacy end points were either of these three: a composite of death from any cause, major coronary event, and nonfatal stroke or a composite of death from CAD, nonfatal MI and urgent coronary revascularization ≥30 days after randomization or a composite of death from CV causes, nonfatal MI, hospitalization for UA, all revascularization ≥30 days after randomization and nonfatal stroke.

**Figure 3:**
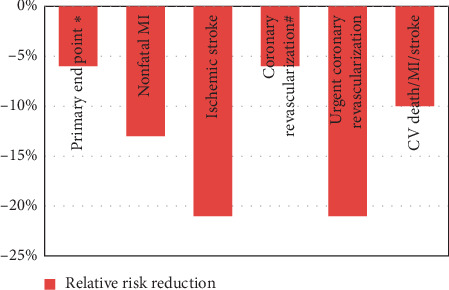
Clinical benefits achieved in the IMPROVE-IT trial (^*∗*^primary end point was a combination of CV death/major CV event and nonfatal stroke; #nonsignificant. There was no difference in CV death and unstable angina).

In this study, the average age of the study population was 64 years, 24% subjects were females, and history of diabetes mellitus was present in 27%. About 88% patients had undergone coronary angiography, while 70% had received percutaneous coronary intervention during the index hospitalization. Only 34% were already on statins before the index event, and 77% received statin therapy during hospitalization. After 1 year of therapy, the mean LDL-C was 69.9 mg/dl in the simvastatin group and 53.2 mg/dl in the simvastatin plus ezetimibe group representing 16.7 mg/dl (24%) further reduction in LDL-C with the combination therapy. At 7 years, the rates for the primary end point were 32.7% in the simvastatin–ezetimibe group and 34.7% in the simvastatin monotherapy group (absolute risk reduction(ARR) of 2.0%; hazard ratio (HR): 0.936; 95% confidence interval (CI): 0.89 to 0.99; *P* = 0.016). The rate of all 3 secondary end-points were also significantly reduced in simvastatin-ezetimibe group (Figure 3). The occurrence of side effects like derangement of LFT, gall bladder diseases, and muscle symptoms were similar in both the groups. The rate of discontinuation of therapy was 10.1% in the simvastatin monotherapy and 10.6% in the simvastatin-ezetimibe group. The benefits of ezetimibe combination to simvastatin were evident in all the subgroups especially in diabetics and those above 75 years of age. The number needed to treat (NNT) for the prevention of one primary end point event was 50.

### 3.1. Ezetimibe in Diabetes Mellitus (DM)

Diabetes mellitus increases the risk of coronary artery disease and the presence of DM in patient presenting with ACS portends poor outcomes [18]. However, interestingly, diabetic patients derive greater benefits compared to their nondiabetic counterparts.

In the prespecified subgroup analysis of IMPROVE-IT, DM was present in 4933 (27%) patients of the study population [19]. Patients with DM were more likely to be female; were older; and had a history of prior MI or CABG. These were less likely to present with an ST-elevation MI (*P* < 0.001) as compared with patients without DM. These patients were more likely to be on guideline-supported therapies and were more likely to be on statins prior to the index event. There was an ARR of 5.5% (HR, 0.85; 95% CI, 0.78–0.94) by combination therapy in diabetic patients at 7 years, while in nondiabetic patients, the ARR was only 0.7% (HR, 0.98; 95% CI, 0.91–1.04; Pint = 0.02). The NNT for primary end point event reduction was 38 as compared to 50 in overall population described above. The greatest relative reductions were due to reduction in MI (24%) and ischemic stroke (39%) in diabetics. There were no differences in safety outcomes in two treatment arms regardless of DM. More importantly, when stratified according to the TIMI risk score, all diabetics irrespective of the risk zone tend to derived benefit, whereas in nondiabetics, only those with high risk had benefit from the ezetimibe combination reiterating DM as a risk modifier.

### 3.2. Ezetimibe and Elderly

In patients presenting with acute coronary syndromes, increasing age is an important factor for adverse prognosis. Although individuals with age >75 years of age represent only <6% of total ACS patients, up to 65% of ACS mortality is seen this cohort [20, 21]. However, use of high dose statin therapy leads to significant adverse effects in elderly. Thus, the combination of ezetimibe to statin might achieve target LDL-C among them without increasing the adverse effects.

It was found in the IMPROVE-IT trial that the combination therapy significantly reduced LDL levels in patients >75 years of age and resulted in risk reduction. The reduction in absolute risk of primary end points in them was higher than young individuals, and the combination was also well-tolerated [22].

### 3.3. Ezetimibe in Chronic Kidney Disease (CKD)

Individuals with CKD are at heightened risk for cardiovascular mortality, and eGFR is an independent and robust predictor of risk [23]. A meta-analysis of 14 statin trials in CKD showed a trend towards reduction in occurrence of first CV event through LDL lowering. However, this benefit decreases as GFR declines further [6].

SHARP was the first trial to show that the major CV events were safely reduced by simvastatin and ezetimibe combination in a wide range of CKD patients [16]. Based on this strong results from this trial, 2013 ACC/AHA guidelines endorsed the use of ezetimibe patients with eGFR <60 ml/min [24]. Data from the IMPROVE-IT study mirrored similar findings [25]. Out of a total 18,144 patients, 3791 had eGFR <60 ml/min. Such patients were more frequently elderly, female, and nonsmoker with additional comorbidities. Addition of ezetimibe improved lipid parameters across all eGFR subgroups. In both the groups, the event rates were higher with lower eGFR. However, the addition of ezetimibe over statins produced a 12% and 13% risk reduction in patients with baseline eGFR <60 ml/min and <45 ml/min, respectively.

### 3.4. Ezetimibe and Plaque Regression on Imaging

Plaque burden is an important surrogate marker of future CV events. Various imaging techniques like IVUS are available to measure plaque volume. Studies have shown that statins modify the natural history of CAD by decreasing plaque progression and may even result in atheroma regression. Although it was evident from the IMPROVE-IT trial that the addition of ezetimibe to statins further lowers LDL-C and, hence, reduces future events especially after ACS, but whether it has any effect on atheromatous plaque was not known.

In the PRECISE-IVUS trial, it was studied that whether aggressive lipid lowering by adding ezetimibe to standard statin therapy has any effect on coronary atherosclerosis [26]. When observed by IVUS examination, an individual who received combination therapy had significant plaque regression (assessed by percentage atheroma volume) compared to monotherapy (78% vs. 58%; *P*=0.04). Subsequently, in a metanalysis of 6 trials of statin and ezetimibe combination therapy, it was found that the combination therapy resulted in more plaque regression [27]. The decline in total atheroma volume from baseline to follow-up was of –3.71 mm^3^ (mean difference, 95% CI-25.98 to 21.44, *P*=0.001), while the percent atheroma volume attenuation stood at a mean of –0.77% (–1.68 to –0.14, *P*=0.10).

### 3.5. Ezetimibe in Post-CABG Patients

Patients with prior CABG are at high risk for recurrent ischemic events as they have extensive atherosclerotic disease. Also, when these patients present with ACS, the prognosis is quite poor [28]. Thus, these patients should receive the best drug treatment for attenuating the atherosclerotic burden in order to improve their prognosis.

In a subanalysis of the IMPROVE-IT trial, it was found that patients of ACS who had prior CABG had poor prognosis having multiple risk factors like older age, prior MI, DM, hypertension, PAD, and prior stroke [29]. Secondly, there was enhanced benefit on adding ezetimibe to statin in such patients which was beyond LDL reduction, possibly due to some pleotropic effects such as inhibition of vascular smooth muscle proliferation, sterol reduction, and antioxidant effects.

### 3.6. Ezetimibe and Gender

LDL lowering by statins has been proven to decrease cardiovascular outcomes in both men and women alike [30]. But, unfortunately there is an inertia among physicians for prescribing statin therapy in females [31]. The role of nonstatin drugs in reducing CV outcomes in females is less clear.

In a prespecified subgroup analysis of IMPROVE-IT, 24% of participants were females [32]. At the study completion, LDL-c was lowered comparably in both the groups (16.7 mg/dl& 16.4 mg/dl in males and females, respectively). Interestingly, despite a similar LDL reduction females had numerically more reduction of primary end point (12% vs. 5% relative risk reduction in men and women, respectively). Although, the results did not reach statistical significance, it will be worthy to note that benefits were primarily driven by amelioration of MI.

### 3.7. Ezetimibe and Recurrent CV Events

In the IMPROVE-IT trial, there were a total of 9,545 primary end point events, and out of them, 56% were first events and rest 44% were subsequent events which were not included in the primary analysis [33]. By virtue of a long follow-up period, up to 13 percent of patients experienced recurrent events in the study. The proportion of unstable angina, stroke, and CV death were not different between first and recurrent episodes. However, there were lesser MI's and higher revascularization in subsequent events as compared to index events.

Ezetimibe-based combination therapy not only reduced first CV events but also second, third, and subsequent events. The additional event reduction in the combination arm with first and recurrent events was −170 (6.2%) and −241 (11.2%), respectively. For every 100 patients treated with ezetimibe-based combination therapy for 10 years, 5 nonfatal MI, 4 revascularizations, and 2 nonfatal strokes are prevented. No CV death and unstable angina events are prevented. Hence, a composite of 11 primary end point events are prevented (Table 2).

### 3.8. Biomarkers and Risk Reduction by Ezetimibe

A multimarker strategy has been shown to be useful for risk stratification and prognosis in ACS patients [34]. Clinical markers have been used to triage patients who are at the highest risk and benefit from the addition of ezetimibe therapy [35]. Additionally, the role of biomarkers in the post-ACS scenario for utilization of ezetimibe therapy has been studied by Qamar et al. [36]. In a subanalysis of the IMPROVE-IT trial, high-sensitivity troponin T, *N*-terminal pro–B-type natriuretic peptide, growth-differentiation factor-15, and high-sensitivity C-reactive protein were estimated in 7,195 patients after 1 month of ACS. Independent associations of each these biomarkers were seen with outcomes of death/MI/stroke and CV death/heart failure. It was also demonstrated in that patients deemed to be at higher risk based on the elevation of biomarkers, the ezetimibe combination therapy was associated with greater absolute risk reduction. A graded response for absolute benefit with ezetimibe was seen with the number of biomarkers elevated (See Table 3).The number needed to treat (NNT) in the cohort with 3 or more biomarker positive was only 14.

### 3.9. Ezetimibe in Statin-Intolerant Patients

Both European and American guidelines recommend statins as first-line lipid-lowering therapy in patients with ASCVD. However, in individuals who have uncontrolled LDL with statins and who are intolerant to statins, there is an emerging consensus on the role of using ezetimibe or proprotein convertase subtilsin/kexin type 9 (PCSK9) inhibitors [37].

Statin intolerance is not uncommon, and according to the observational studies, approximately 25% of the patients have some degree of statin intolerance which may lead to nonadherence [38]. Nonadherence to statin is not benign and increases the risk of recurrent MI and coronary heart disease events [39]. In a simulation analysis by Canon et al., it was found that the use of ezetimibe is increased by 8% while that of PCSK9 inhibitors by 7% in patients having partial or full statin intolerance (assumed at 10%) [40]. However, PCSK9 inhibitors have cost issues, and it may itself lead to drug discontinuation, so ezetimibe is a first-line option in such patients being an effective lipid-lowering drug with low cost [41].

### 3.10. Ezetimibe and Drug Adherence

Premature discontinuation of lipid-lowering therapies is a major impediment in achieving secondary prevention goals after acute coronary syndromes [42]. For statin therapy, on long-term follow-up, the observed discontinuation rates may be as high as 50% [43].

In the IMPROVE-IT trial, the medication discontinuation rate was slightly higher in the simvastatin monotherapy arm vis-a-vis simvastatin plus ezetimibe combination arm (52% vs. 49.8%; *P* = 0.049).Factors favoring drug discontinuation were smoking, prior revascularization, hypertension, unstable angina, female sex, nonwhite race, and US location [44]. The I-ROSETTE study results resonated similar findings [45]. In the study, the combination of ezetimibe to rosuvastatin significantly improved lipid profile (LDL reduced by 50%) in hypercholesterolemia patients without any extra side effects. In fact, the combination was better tolerated as compared to an equivalent dose of rosuvastatin due to different metabolic route of action of the two drugs [45]. So, we can reasonably infer that the combination of statin and ezetimibe has got comparable safety and tolerability as statin monotherapy.

## 4. Guideline Track

According to 2018 ACC/AHA lipid guidelines, for patients with clinical ASCVD who are deemed to be at high risk, an LDL-C goal of <70 mg/dl or >50% reduction in LDL-C from baseline is recommended [46]. However, the latest ESC guidelines for the management of dyslipidemia recommends LDL-C reduction of >50% from baseline or LDL-C <55 mg/dl in them for secondary prevention [47]. In either case, if these goals are not achieved by maximum tolerated doses of statins, addition of ezetimibe is a class IIa recommendation by the ACC/AHA guidelines, while the ESC guidlelines suggest it is a Class I recommendation to add the drug.

## 5. Summary

**Figure 4:**
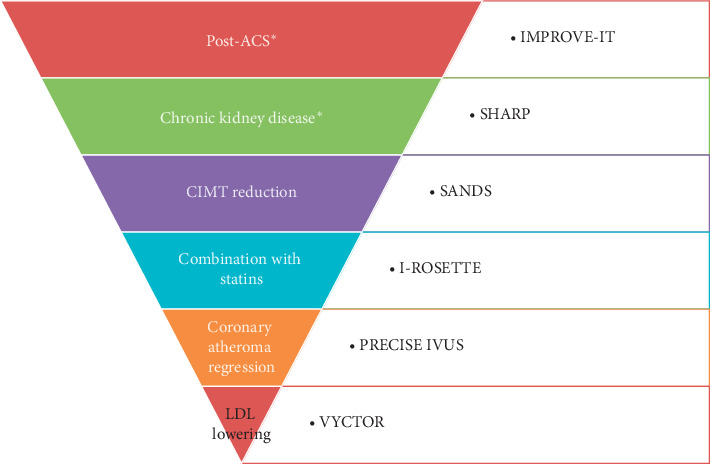
Various clinical scenarios and the corresponding studies where ezetimibe has been shown to be useful. The trials are arranged in a declining order of sample size with the maximum in IMROVE-IT and minimum in VYCTOR. ^*∗*^ Cardiovascular outcome trials (ACS- acute coronary syndrome; CIMT- carotid intima medial thickness).

LDL-C has a pivotal role in the pathogenesis of atherosclerosis and ASCVD. Statins are the primary and most important hypolipidemic agents which reduce LDL along with reduction in future CV events. However, many patients are not able to tolerate high statin doses or have uncontrolled LDL-C even with maximally tolerated doses leading to residual CV risk. Ezetimibe is a cholesterol absorption inhibitor with potent cholesterol lowering effects. As an individual agent, it failed to make an impact initially. The IMPROVE-IT was the game-changing study which pitchforked the drug into limelight. The combination of ezetimibe and statin was found to be effective in producing additional LDL-C reduction on top of statins and reduction of CV events as well. The drug has been effective in a wide range of patient profile too (Figure 4). Above all, the combination is safe and well-tolerated owing to a different mechanism of action on metabolic pathways. The drug has now found a widespread acceptability as an add-on therapy, and the latest guidelines recommend adding ezetimibe if LDL-C targets are not met with statins and there is a residual CV risk.

## Figures and Tables

**Table 1:** Pivotal trials of ezetimibe.

Trial	Year	Number of patients	Study population	Study design	Results
SANDS	2008	499	American-Indian men and women aged 40 years or older with type 2 diabetes and no prior CVD events	Randomized to aggressive (LDL-C < 70, SBP < 115 mmHg) (*n* = 252) vs. standard (LDL-C < 100, SBP < 130 mmHg) (*n* = 247) treatment	CIMT regressed in the aggressive group and increased in the standard group (−0.012 mm vs. 0.038 mm; *P* < 0.001); carotid arterial cross-sectional area was also reduced (−0.02 mm (2) vs. 1.05 mm (2); *P* < 0.001)

ENHANCE	2008	720	Patients of heterozygous FH were taken to see atherosclerosis regression with ezetimibe	Simvastatin 80 mg (*n* = 363) or simvastatin 80 mg plus ezetimibe 10 mg (*n* = 357)	Ezetimibe plus simvastatin did not produce a significant reduction in carotid IMT despite the further reduction in LDL-C and hs-CRP achieved with this drug

ARBITER 6	2010	315	Patients of CAD/CAD equivalent with LDL-C <100 mg/dl and HDL-C <50 mg/dl for men or 55 mg/dl for women (on statin treatment) for CIMT progression	Ezetimibe (10 mg/day) or extended-release niacin (target dose, 2,000 mg/day)	Patients on niacin (*n* = 154) had significant regression in both mean CIMT (−0.0102 ± 0.0026 mm; *P* < 0.001) and maximal CIMT (−0.0124 ± 0.0036 mm; *P*=0.001). Ezetimibe (*n* = 161) did not reduce mean CIMT (−0.0016 ± 0.0024 mm; *P*=0.88) or maximal CIMT (−0.0005 ± 0.0029 mm; *P*=0.88) compared with baseline

VYCTOR	2009	90	To see effect aggressive lipid lowering on CIMT, LDL-C and hs-CRP in high-risk patients	90 high‐risk CAD were allocated to 3 groups: pravastatin 40 mg, simvastatin 40 mg, and simvastatin 20 mg with ezetimibe 10 mg	After 1 year of therapy, a significant reduction in LDL-C to a mean level of 45–48 mg/dL was seen with a significant reduction in all three groups and CIMT values were 0.93 ± 0.13 mm, 0.90 ± 0.11 mm, and 0.92 ± 0.01 mm for groups 1, 2, and 3, respectively

SHARP		9270	9270 patients with CKD	Simvastatin/ezetimibe 20/10 vs. placebo	LDL-C lowering with combination therapy reduced major atherosclerotic events in a wide range of CKD patients

SEAS	2008	1873	Patients of asymptomatic AS	Double blind randomized control trial between simvastatin/ezetimibe 40/10 vs. simvastatin 40 mg on cardiovascular outcomes	No effect on AS progression with LDL-C lowering. There were fewer ischemic cardiovascular events in the combination therapy

IMPROVE-IT	2010	18144	High-risk post-ACS patients	Randomized to simvastatin/ezetimibe 40/10 vs. simvastatin 10 mg	Simvastatin/ezetimibe combination superior to simvastatin monotherapy in reducing events (32.7 vs. 34.7%, *P*=0.016)

**Table 2:** Number of adverse CV events prevented with ezetimibe when added over and above statins (data from the IMPROVE-IT study).

Event type	Numbers halted^*∗*^
Combination of CVD, nonfatal MI,UA, revascularization, and nonfatal stroke	11
Nonfatal MI	5
Nonfatal stroke	2
Revascularization	4

^*∗*^For 100 patients treated for 10 years (CVD- cardiovascular death; UA- unstable angina; MI- myocardial infarction).

**Table 3:** Graded risk reduction by ezetimibe therapy with absolute number of biomarkers elevated in substudy of the IMPROVE-IT trial. Modified from Qamar et al. [36] (NNT- number needed to treat).

Risk category	Number of biomarkers elevated	Absolute risk difference (%)	NNT
High risk	>3	−7.3	14
Intermediate risk	1-2	−4.4	23
Low risk	0	+3.0	—
